# Ointments and Pastes

**Published:** 1892-01

**Authors:** Ernest Wende

**Affiliations:** Clinical Professor of Dermatology, University of Buffalo


### OINTMENTS AND PASTES.

### A CLINICAL LECTURE DELIVERED AT THE FITCH DISPENSARY.

By ERNEST WENDE, M. D., B. Sc.
Clinical Professor of Dermatology, University of Buffalo.

Gentlemen—Allow me, in a passing way, to call your attention to ointments and pastes, those preparations which we so frequently prescribe. The end and aim of all preliminary study of skin diseases, the treatment pursued, has been greatly modified. France no longer resorts exclusively to bitters and sulphur, England no longer to purgatives and various forms of mercury, tortured by ingenuity, and even America has lost faith in sarsaparilla, her once approved and universal specific.

The words of Dante, written over the gateway of hell, "Leave hope behind, all ye who enter here," were first erased from the propylon of the dermatological clinic at Vienna. It was notably Hebra who objected to the orthodox mode, and found that the skin, like other organs, may be idiopathically diseased.

Thus a careful selection of local remedies is generally recognized to promote therapeutic success in the treatment of cutaneous affections. Experience has shown that such a choice is of greater significance than a well-directed constitutional administration of drugs.

Ointments, which are fatty mixtures of medicinal agents, constitute the usual and by far the most useful means for topical application. We have chiefly entering into the composition of their bases some unctuous substance, as lard, beef, or mutton suet, a fixed oil, with more or less wax, or some ingredient like glycerine, vaseline or paraffine having a similar consistence, and lastly, lanoline, a product prepared from sheeps' wool by a process of centrifugation.

The following properties are essential for a good ointment basis:

I. *Proper Consistency.*—It must be soft, smooth and pliable, readily admitting of a uniform application.

II. *Homogeneity.*—It must be homogeneous, perfectly free from grittiness or irritating bodies, hard and crystalline.

III. *Durability.*—It must *not* show a tendency to change its physical and chemical peculiarities on exposure or long keeping.

IV. *Miscibility.*—It must be capable of easily receiving the medicating ingredients to be combined or incorporated, in order to facilitate their absorption and action.

V. *Power of Imbibition.*—It must be capable of absorbing liquids, especially water, the importance of which can be best realized when a salt requires solution before being incorporated, as the iodide of potassium, etc.

VI. *Limitation of Temperature.*—It must have a melting point somewhat higher than the temperature of the body. It may soften and become pliable, but must not liquify.

VII. *Inability to Produce Irritation.*—It must be perfectly bland and neutral in reaction.

*Adeps or Lard* is the purified fat taken from the abdominal cavity of the hog, its specific gravity being about 0.935, its melting point 95° F., and it is readily soluble in ether, benzine and the disulphide of carbon. We will find that lard, after long preservation, on exposure to the light and air, especially a damp atmosphere, is apt to become rancid. It absorbs oxygen freely, and is readily subjected to decomposition, forming glycerine and volatile acids. Besides, when under the influence of water in compounding ointments, rancidity is easily acquired. The aquisition of an acid reaction, no matter how slight, often increases the irritation of the diseased parts, which complicates the reparative process and retards recovery. Again, the affinity of lard for water is but trifling, and it assists but sparingly in the absorption of remedies by the skin. In fact, it is only fit for pharmaceutical purposes when fresh.

Vaseline or petroleum fats are substances prepared from the waste products of petroleum distillation, having a melting point which varies from 104° to 125° F.

They are insoluble in water, sparingly so in alcohol, and quite soluble in ether, chloroform, disulphide of carbon, oil of turpentine, benzine, benzol, and in fixed and volatile oils. Vaseline and its allied class have the great advantage of being durable and not prone to turn rancid, while the disadvantages are their incompatibility with water, their inferior penetrating powers, and their capability, in a few instances, where the skin is delicate and sensitive, of producing irritation.

Suet, spermaceti, wax, cacao-butter and paraffine, are too hard on the one hand, while glycerine and the fixed oils are too soft on the other, in themselves to form a good ointment basis. They are simply and occasionally used as ingredients, the former to impart firmness, and the latter pliability.

Lanoline or wool-fat being a cholesterine fat, a true product of keratinous tissues, it is but natural to expect it to possess those qualities absolutely necessary for an excellent ointment basis. As a derivative from sheeps' wool, it was brought to notice by Prof. Liebreich, of the University of Berlin.

It possesses a specific gravity of about 0.935, while its melting point fluctuates between 100.4° and 104° F. It is soluble in ether, benzol, benzine, chloroform and disulphide or carbon, slightly in strong alcohol and insoluble in water.

It is unoxidizable, non-irritant and neutral in reaction. It is readily absorbed and retained by the skin, increasing the therapeutic action of the drugs incorporated. It will readily imbibe more than its own weight of an aqueous solution without losing its pliability or salve-like form. It adheres better to a moist surface than any other fat or oil. It facilitates union with glycerine, and is easily miscible with all other fats and oils. It is germtight, irreducible and hermatically sealed to microörganisms.

Moreover, its shortcomings are few and not grave, being merely charged of having a consistency slightly sticky, and a faint, sheep-like odor.

It occurs in the shops in two forms, one containing about twenty-five per cent. of water, the other free from it. The anhyrous lanoline, in my judgment, makes the much better preparation, for the addition of water might irritate in some instances, and whenever it becomes necessary to incorporate water or an aqueous solution it can be readily added at any time. To overcome the slight stickiness, I generally combine with it ten to twenty per cent. of vaseline.

Ointments may be considered as :

I. *Sedative*, when they counteract an inflammatory process, cool the burning and soothe the irritation. They act mostly in protecting the diseased surface from the stimulating action of the air and moisture, and comprises the simple ointments, cold cream, lanoline cream, etc.

II. *Astringent*, when they counteract a lax condition of the skin. They are also soothing in their action. They act in modifying inflammation and secretion by constricting the calibre of the capillaries and the ducts of glands. They include such substances as zinc, bismuth, lead, tannin, etc.

III. *Stimulating*, when they counteract a morbid process of the skin by setting up a new action, hastening nutritive changes, which re-establish the normal performance of its functions. They contain such ingredients as sulphur, tar, mercury, naphthol, resorcin, etc.

IV. *Antiseptic* when they counteract infection. They act by preventing putrefaction and fermentation, inasmuch as they destroy microörganisms, restricting their growth and multiplication. They comprehend such remedies as iodoform, iodol, aristol, corrosive sublimate, carbolic acid, etc.

V. *Antiparasitic*, when they counteract the life, growth, and development of the various low forms of animal and vegetable life infesting the human epidermis. They act by producing direct impressions, which are poisonous to the parasite. Among them may be found styrax, beta-naphthol, mercury, balsam Peru, sulphur, etc.

VI. *Antipruritic*, when they counteract itching. They act in all probability, by paralyzing the peripheral nerves, inducing, locally, an anesthesia which relieves the over-excitation of the sensitive nerves, or by modifying an abnormal secretion or excretion, They include such remedies as carbolic acid, terebene, menthol, camphor, cocaine, etc.

Ointments are curative agents, their functions being to protect the diseased skin from all external irritants, and impurities from atmospheric influences and vicissitudes ; to aid in the absorption of medicaments ; to relieve by cooling and soothing the burning sensation of inflammation ; to mitigate pain ; to allay itching ; to alleviate an unusual dryness and harshness ; to astringe capillaries in serous effusions ; to loosen and remove an accumulated débris of crusts ; to resolve effusions, infiltrations, and new formations ; to render the parts aseptic and impassable to germs ; and, furthermore, to regain and preserve the integrity of the integument by stimulating nutrition.

### PASTES.

Pastes, during the last few years, have been largely used in the treatment of skin affections, especially for the various types of eczema. They were first introduced to the notice of the profession by Lassar and Unna, of Germany. They are applications which resemble ointments having a firmer consistence, however, due to a powder incorporated in an unusual proportion. The substances used may be either of a mineral or vegetable origin. The minerals generally employed are talc, bolus alba, chalk, zinc oxide, calcium and magnesium carbonate, while those taken from the vegetable world are usually starch and lycopodium.

The advantages over ointments are twofold. They not only defend the eruption from external irritation, exclude the air, preventing desiccation and oxidization, but the excretions and secretions of the diseased part are also absorbed. Furthermore, they aid adhesion, producing a fixation of medicaments.

Of these the one that has enjoyed the best reputation is that first prescribed by Oscar Lassar, and named after him. It consists of

℞.—Acidi salicylici ........................ 2.00
Zinci oxidi ............................
Amyli ..................................
Vaseline ...............................
Lanolini anhydr. aa .................. 25.00

Misce. Leniter lerenda fiat pasta.

This exceedingly useful therapeutical preparation is of extensive applicability, notably in the different forms of eczema.

Ihle's paste contains resorcin in lieu of salicylic acid, the formula being—

℞.—Resorcini ........................ 2.00 to 12.00
Zinci oxidi ............................
Amyli ..................................
Vaseline ...............................
Lanolini anhydr. $\overline{aa}$ .................. 25.00

Its special indications are for employment as a reducing agent. As Unna puts it, when topically applied it acts "as a kerato-plastic agent. It makes the swollen and defective horny layer harder, thicker, and drier, so that it may again become more fit to take up fat."

A paste which we have found most soothing and bland in the treatment of infantile eczema, in modifying a simple dermatitis or in allaying irritation, is composed of—

℞.—Campho-phenique .................... 1.00
Bismuthi subnit ........................
Zinci carbonatis .......................
Amyli ..................................
Vaseline ...............................
Lanolini anhydr. $\overline{aa}$ .................. 2.00

Other remedies as tar, sulphur, beta- and hydro-naphthol, and ichthyol may be advantageously combined.

Their mode of application is a matter for attention and of importance. They should be gently rubbed upon the diseased part with the fingers or an appropriate spatula, the former being used when the skin is tender. They should be well introduced in all cracks and crevices, and uniformly distributed in a moderately thick layer over the whole of the affected epidermis, in order that the physiological and therapeutical properties possessed by a paste may be justly fulfilled, namely :

The fixation of the medicaments incorporated.

The absorption of secretion and excretion of the lesion ; and the protection of the diseased integument.

